# Cost-Sharing Effects on Hospital Service Utilization Among Older People in Fukuoka Prefecture, Japan

**DOI:** 10.34172/ijhpm.2020.190

**Published:** 2020-10-13

**Authors:** Yunfei Li, Akira Babazono, Aziz Jamal, Peng Jiang, Takako Fujita

**Affiliations:** ^1^Department of Health Care Administration & Management, Graduate School of Medical Sciences, Kyushu University, Fukuoka, Japan.; ^2^Health Administration Program, Faculty of Business & Management, Universiti Teknologi MARA, Selangor, Malaysia. 3; ^3^Department of Health Sciences, Faculty of Medical Sciences, Kyushu University, Fukuoka, Japan.

**Keywords:** Cost-Sharing, Hospitalization Cost, Length of Stay, Older People, Japan

## Abstract

**Background: **The cost-sharing impact on hospital service utilization of different services is a critical issue that has not been well addressed worldwide. This study aimed to investigate the cost-sharing effects based on income status on hospital service utilization of different services among elderly people in Japan and provide a comprehensive examination and discussion for the reasonability of a cost-sharing system.

**Methods: **The data were extracted from the Latter-Stage Elderly Healthcare Insurance database in the fiscal year 2016. A total of 610 182 insured people aged ≥75 years old, with 155 773 hospitalization patients, were identified. Hospitalization rate, length of stay (LOS), and total hospitalization cost were used to test the statistical significance among patients categorized by income levels. Generalized linear models for total hospitalization cost were constructed based on bed types to further assess different hospital service utilization.

**Results:** For medical chronic care and psychiatric beds, which both required long-term care treatment, much higher hospitalization rates were observed in the patients with low- and middle-income levels than patients with high-income level. The LOS and total hospitalization cost of the patients with low- and middle-income levels were significantly higher than the patients with high-income level treated in medical chronic care and psychiatric beds. For psychiatric beds, the total hospitalization cost for patients with low-income level was significantly higher than that for patients with highincome level.

**Conclusion:** The cost-sharing policy in Japan, especially the cap for out-of-pocket needs further determination. The importance of community-based care services needs to be emphasized, and the collaboration between hospitals and community-based care facilities should be enhanced.

## Background

**Key Messages**

Implications for policy makers

- A change of policy to reduce the cap for out-of-pocket would discourage patients and caregivers from using hospital admission service as a substitute for long-term elderly care.
- Public campaigns regarding the need for sustainable community-based care services could be regularly organized to increase people’s awareness, and consequently support the utilization of community-based healthcare services.
- An appropriate hospital admission guideline targeting long-term care treatment could be issued. An enhanced collaboration among hospitals, long-term care facilities and other entities providing community-based support services is needed.

Implications for the public

Cost-sharing policy in Japan is not all-inclusive despite decades of effort. The current cost-sharing policy for older people provides incentives for a large number of “social hospitalization” cases. Public, in general, must aware that hospital services are for medically necessary conditions and not to be used as substitutes for the long-term care that provides living and support services. The increase of co-payment cap would necessarily impact on the out-of-pocket payment for long hospital stays, hence preventing patients from staying in the hospital longer than it is medically needed. Community-based care facilities could play an important role to promote the proper use of long-term care services for older people.

Japan is struggling with a growing aged population. In 2016, the number of people aged 75 years or more was 16.91 million, which was 13.3% of Japan’s total population.^1^ With this increase in the number of older people in Japan, a dramatic increase in healthcare demand due to age- and lifestyle-related diseases is expected. However, healthcare costs and utilization are varied according to the hospitalization bed types.^2-4^ A case–mix system called Diagnosis Procedure Combination (DPC) was developed to increase the efficiency and provide a comprehensive care service for inpatients.^5^ Using the DPC system, hospitalization bed types can be categorized further into DPC beds, general beds other than DPC (named as general beds), medical chronic care beds, psychiatric beds, and others. Based on a survey conducted by the Japanese Ministry of Health, Labor and Welfare in 2016, the hospitalization cases according to general, medical chronic care, psychiatric, and other hospital beds, were 891 398 (57.1%), 328 161 (21%), 334 258 (21.4%), 7188 (0.5%), respectively.^6^

 Pressure on the Japanese government to control healthcare spending and efficiently allocate medical resources led to changes in cost-sharing policies for older people. Through several changes and a universal healthcare coverage plan in Japan,^7^ a cost-sharing system targeting older people called “Latter-Stage Elderly Healthcare Insurance” was implemented, which consists of a co-payment rate and a cap for out-of-pocket cost. Since April 2008, residents aged 75 and over with low- and middle-income levels were assigned to a 10% co-payment rate, whereas the co-payment rate for high-income residents (comparable to the workforce standard) was set at 30%. In addition, the coverage for medically catastrophic conditions stipulates a monthly cap for out-of-pocket cost to financially assist patients based on their income levels. For patients with low-income level, the out-of-pocket for all medical costs were capped monthly at JP¥ 24 600 (US$223.64) or JP¥ 15 000 (US$136.36). For patients with middle-income level, the costs were capped monthly at JP¥ 44 400 (US$403.64). For patients with high-income level, the costs were capped monthly at JP¥ 80 100 (US$728.18) and a 1% co-payment rate applies above this cap.^8,9^

 Despite the government effort in its attempt to provide equitable healthcare, the current co-payment rates and cap replacement for out-of-pocket expenses are not without criticisms. A substantial increase in hospitalization rate along with the costs for providing care—especially among low-income patients—were evident across literature, leading some critics to dub this phenomenon as “*social hospitalization*.” This phenomenon is unique to Japan, where patients or caregivers use hospital admission as a substitute for receiving care in long-term care facilities due to cheaper out-of-pocket cost.^10,11^ Although it is never been fully investigated, the preference of patients or caregivers to use hospital admission might also be attributed to the perception that hospital provides superior medical care than what they could get from the long-term care facility. This perception, however, is not entirely true as most long-term facilities in Japan are quality-certified, sufficiently equipped, and housed by trained medical personnel. Besides that, a relaxed admission policy at the hospital level might also explain the increasing number of hospitalized patients with chronic conditions who are better treated in a long-term care facility.

 In Japan, the cost-sharing policy is uniform regardless of the hospitalization bed types when healthcare services are utilized.^12,13^ This study aimed to provide a comprehensive analysis of the impact of the cost-sharing policy on healthcare utilization among older people by calculating hospitalization rate, length of stay (LOS), cost per patient per day, and total hospitalization cost as categorized by income levels. Therefore, the healthcare utilization of four bed categories, ie, DPC beds, general beds, medical chronic care beds, and psychiatric beds, was analyzed separately. The study findings might be able to assist relevant stakeholders and policy-makers in determining reasonable and equitable healthcare allocations for older people in Japan.

## Methods

###  Data Source

 This study was conducted using healthcare claims data provided by the Wide-Area Association of Latter-Stage Elderly Healthcare of Fukuoka Prefecture, Japan. The Latter-Stage Elderly Healthcare Insurance is an insurance scheme designed specifically for older residents aged 75 years or older and residents aged between 65–74 years old with a specified disability. The participants’ ID, gender, birth date, diagnostic information, medical cost information, and treatment procedure are available in the claims database.

 This study used data extracted from April 1, 2016 to March 31, 2017. The claims database was used to identify the insured people whose personal identification numbers were valid during the period of April 1, 2016 until March 31, 2017. Information including sex, age, patients’ income categories, healthcare use, and relevant medical cost information were extracted from the database. The collected information regarding healthcare use during the 1-year period included admission frequency, LOS, and the amount of healthcare costs billed to the insurance provider. We used a conversion rate of JP¥ 110 to US$1.

 To assess the severity of comorbidity status, the Charlson Comorbidity Index (CCI) was used. This instrument was widely used in Japan.^14,15^ The use of this instrument in the previous study showed that the index has acceptable reliability, thus could be applied to studies analyzing healthcare data from the Japanese insurance claims database.^16^ The International Classification of Diseases 10th Revision (ICD-10) coded data were used to capture patients’ morbidity status. CCI was used to identify 17 reported comorbidities (acute myocardial infarction, congestive heart failure, peripheral vascular disease, cerebral vascular disease, dementia, chronic pulmonary disease, connective tissue disorders, peptic ulcer, liver disease, diabetes, diabetes complications, paraplegia, renal disease, cancer, metastatic cancer, severe liver disease and HIV).The CCI has been used in many settings in Japan,^16^ and the assessment of reliability of CCI indicated satisfying discriminatory ability. This instrument assigns weights ranging from 1 to 6 the presence of comorbidity. The CCI scores for each patient were calculated by adding all comorbidities’ weights. Because hypertension is one of the most common chronic disease in Japan and excluded from CCI assessment, patient’s hypertensive disease status (ICD-10 code: I10-I15) was also extracted individually.

 The database also provided information on hospitalization bed types where either DPC beds or non-DPC beds were required for each hospitalization episode. According to the categorization of the basic hospitalization fees, admission receipt codes were subsequently used to identify non-DPC admission groups, ie, general beds other than DPC, medical chronic care beds, and psychiatric beds. SQL Server 2014 was used to extract the data from the database.

 Most of these databases were computer-administered with a high penetration rate, which was as much as 98.6% until April 2015 according to a Japanese government report.^17^ Japanese Health Insurance Claims Review & Reimbursement services are responsible for the quality control of computer-administered claims databases.

###  Study Population

 All individuals residing in Fukuoka Prefecture during the 2016 fiscal year aged 75 years and older with admission records were regarded as the study subjects. Because the fiscal year in Japan begins on 1st April and ends on 31st March annually,^18^ the 1-year retrospective cohort study data were extracted from April 1, 2016 until March 31, 2017.

###  Definition of Variables

 Basic information, namely sex and age were extracted from the claims database. Age was categorized into three groups: ie, 75 to 79 years old, 80 to 84 years old, and ≥85 years old. The CCI scores were used to categorize three levels of health status: mild (CCI = 0–1), moderate (CCI = 2–3), and severe (CCI = 4–higher).^19^ Individuals’ hypertension status also identified to assess patients’ chronic disease status.

 Income levels were classified into three groups: ie, low-, middle-, and high-income levels. Since co-payment rates and caps vary with income levels, calculation of marginal rates of co-payment (once the cap was exceeded) might not be possible. Therefore, we calculated the actual average cost-sharing percentage for each income level. The actual average cost-sharing percentage was calculated by dividing each patient’s out-of-pocket expenses, with each patient’s total medical expense. Total medical expenses were extracted from the database. Calculated total medical expenses include outpatient cost, hospitalization cost, and medication cost. Out-of-pocket expenses were calculated based on the information presented in Table 1. The actual data for the one-year study period, however, indicate that the average cost-sharing percentages were 24.25% (high-income), 8.04% (middle-income), and 6.33% (low-income). Similar calculation method was also applied in the previous study.^20^ We, therefore, assume these rates reflect the co-payment rates applied to patients without a specific cap.

**Table 1:** Cost-Sharing Schedule for the Elderly Over 75 in Japan

	**Co-payment Rate (%)**	**Cap for Out-of-Pocket Cost Per Month (JP¥)**
High-income	30	80 100 + (total medical expense – 267 000) × 1%
Middle-income	10	44 400
Low-income	10	24 600 or 15 000 (depending on income)

 According to medical care function, medical institutions were classified into four types: DPC beds (beds providing comprehensive care services for inpatients based on DPC), general beds other than DPC (beds with nursing workforce allocation), medical chronic care beds (beds for the patients that need long-term medical treatment), and psychiatric beds.

 To calculate hospitalization rate, the total number of hospital admissions was divided by the total number of the insured people of Fukuoka Prefecture during a specific fiscal year. The LOS in this study refers to the duration of hospitalization for any medical condition requiring care and was calculated by dividing the total number of days hospitalized with the number of admissions a patient had in a fiscal year. However, total hospitalization cost refers to the hospitalization cost incurred by each person during the fiscal year. For a better estimation, the cost per patient per day was also calculated by dividing total cost with LOS for each bed type.

###  Data Analysis

 Descriptive analyses for the insured people and inpatients were conducted to examine the distributions of sex, age, CCI score, and hypertension based on income levels. To evaluate the influence of different income levels on hospitalization rate, separate hospitalization rate was calculated for low-, middle-, and high-income levels in four bed-type categories.

 As for the evaluation of hospital utilization, the data regarding LOS, cost per patient per day, and total hospitalization cost were analyzed using descriptive statistics and presented as mean and standard deviation values. The categorical variables of income levels were statistically compared using Kruskal–Wallis tests because the healthcare utilization data are nonnormally distributed.

 Finally, to determine the influence of sex, age, CCI, and hypertension, on total cost, analyses using a generalized linear model were performed on each bed type. In these analyses, total hospitalization cost was set as the dependent variable, and sex, age, CCI, hypertension, and income levels were set as independent variables. The applied generalized linear models assumed gamma distribution, which provides a superior approach when dealing with skewed data distribution.^21^ The fitted model also incorporated a log link that assumed a multiplicative or proportional effect. Therefore, the results provided estimates for the cost ratio. *P* < .01 were regarded as statistically significant. All statistical analyses for this study were carried out using Stata statistical software, released 14.0 (Stata Corp, College Station, TX).

## Results

###  Descriptive Analysis

Table 2 shows demographic information of the insured people. A total of 610 182 insured people were identified from the database. The great majority belong to the middle- (n= 302 558, 49.58%) and low- (n= 276 861, 45.27%) income levels. Patients with high-income level comprised only about 5% (n= 30 763) of the total number of insured people. The number of females (n= 386 705, 63.38%) was higher than the number of males (n= 223 477, 36.62%). As for CCI scores, the number of people with mild severity was 322 547 (52.86%) among the study subjects. The proportion of all insured people with records of hypertension was 66.02% (n= 402 827).

**Table 2:** Demographic Characteristics of Insured People

	**Low-Income Level**	**Middle-Income Level**	**High-Income Level**	**Total (%)**
Total	276 861	302 558	30 763	610 182 (100)
Gender				
Male	61 774	144 852	16 851	223 477 (36.62)
Female	215 087	157 706	13 912	386 705 (63.38)
Age				
75–79	82 235	110 911	13 308	206 454 (33.83)
80–84	84 396	97 149	9498	191 043 (31.31)
≥85	110 230	94 498	7957	212 685 (34.86)
CCI score				
Mild	149 689	157 385	15 473	322 547 (52.86)
Moderate	81 752	89 663	9202	180 617 (29.60)
Severe	45 420	55 510	6088	107 018 (17.54)
Hypertension	180 678	201 899	20 250	402 827 (66.02)

Abbreviation: CCI, Charlson Comorbidity Index.

###  Results from the Analyses of the Variance in Healthcare Utilization

Table 3 shows the healthcare utilization among different income levels according to bed types. The hospitalization rate was calculated statistically. Within a 1-year period, the total hospitalization rate was 34.28%. The hospitalization rate for patients with low-, middle-, and high-income levels were 36.02%, 32.97%, and 31.52%, respectively. In comparing the bed types, the highest hospitalization rate was for DPC beds (total = 19.01%) with a low-income level of 18.72%, middle-income level of 19.21%, and high-income level of 19.60%, while the lowest hospitalization rate was for psychiatric beds (total = 0.80%). The hospitalization rate for psychiatric beds for patients with low-income level was 1.05%, for the middle-income level was 0.61%, and for the high-income level was 0.40%. For DPC and general beds, hospitalization rates were almost the same among each income level. However, for medical chronic care and psychiatric beds, the hospitalization rate for low-income levels was much higher than that for the middle- and high-income levels.

**Table 3:** Healthcare Utilization Between Different Income Levels According to Hospitalization Bed Types

	**DPC Beds**			**General Beds**			**Medical Chronic Care Beds**			**Psychiatric Beds**		
	**Low-Income Level**	**Middle-Income Level**	**High-Income Level**	**Low-Income Level**	**Middle-Income Level**	**High-Income Level**	**Low-Income Level**	**Middle-Income Level**	**High-Income Level**	**Low-Income Level**	**Middle-Income Level**	**High-Income Level**
Hospitalization rate												
(%)	18.72	19.21	19.60	10.97	9.59	9.36	5.28	3.56	2.16	1.05	0.61	0.40
Total (%)	19.01			10.20			4.27			0.80		
LOS (days)												
Mean	18.9	18.5	17.5	33.0	30.6	28.1	123.6	108.5	94.1	160.1	130.9	92.0
SD	16.6	16.9	16.8	38.0	36.1	35.7	112.9	103.6	95.3	135.0	121.6	99.7
Max	234	204	183	365	365	365	365	365	365	365	365	365
χ^2^	119.87			149.37			102.90			62.35		
*P* value	<.01			<.01			<.01			<.01		
Cost per patient per day (US$)												
Mean	556.04	581.25	613.12	336.41	356.02	401.65	176.89	182.34	191.55	155.91	165.40	175.61
SD	453.60	537.24	521.20	289.54	273.60	384.02	47.64	46.71	52.01	49.03	61.30	73.14
χ^2^	145.93			287.56			135.69			94.23		
*P* value	<.01			<.01			<.01			<.01		
Total hospitalization cost (US$)											
Mean	9134.72	9201.92	9091.37	8836.47	8606.67	8437.87	20 691.54	18 825.85	16 825.35	21 956.47	18 770.67	13 574.12
SD	8782.31	9252.28	9229.81	8996.47	9214.47	9351.00	19 542.97	18 473.57	17 250.54	17 223.75	15 806.54	13 366.68
χ^2^	21.16			37.16			62.12			50.11		
*P* value	<.01			<.01			<.01			<.01		

Abbreviations: LOS, length of stay; DPC, Diagnosis Procedure Combination; SD, standard deviation.

Table 3 also shows the differences in LOS among income levels according to bed types. Patients with low-income levels generally had a statistically longer LOS than patients with high-income levels. The LOS of the low-, middle-, and high-income levels were significantly different for each bed type when tested using Kruskal–Wallis tests: ie, DPC beds (χ^2^ = 119.87, *P *<.01), general beds (χ^2^ = 149.37, *P* <.01), medical chronic care beds (χ^2^ = 102.90, *P* <.01), and psychiatric beds (χ^2^ = 62.35, *P* <.01). The longest period for hospitalization was 365 days for general, medical chronic care, and psychiatric beds. For total hospitalization cost, significant differences among low-, middle-, and high-income levels were observed for each bed type: ie, DPC beds (χ^2^ = 21.16, *P* <.01), general beds (χ^2^ = 37.16, *P* <.01), medical chronic care beds (χ^2^ = 62.12, *P *<.01), and psychiatric beds (χ^2^ = 50.11, *P* <.01).

###  Multivariable Analysis of Bed Types

 Analyses using a generalized linear model with log link and gamma distribution were conducted on total hospitalization cost for each bed type by fitting a linear combination of sex, age, CCI score, and hypertension as predictor variables (Table 4).

**Table 4:** Generalized Linear Models With Log Link Function and Gamma Distribution for Prediction of Total Cost According to Hospitalization Bed Types

	**DPC Beds**		**General Beds**		**Medical Chronic Care Beds**		**Psychiatric Beds**	
	_e_ **β**	**(95% CI)**	_e_ **β**	**(95% CI)**	_e_ **β**	**(95% CI)**	_e_ **β**	**(95% CI)**
Gender								
Male	Reference							
Female	1.01	(1.00–1.03)	1.05*	(1.03–1.07)	1.03	(1.01–1.06)	1.07*	(1.02–1.13)
Age								
75–79	Reference							
80–84	1.00	(0.98–1.01)	1.01	(0.98–1.03)	0.90*	(0.87–0.94)	0.97	(0.91–1.03)
≥85	0.96*	(0.94–0.97)	1.02	(1.00–1.04)	0.83*	(0.80–0.86)	0.90*	(0.84–0.95)
CCI score								
Mild	Reference							
Moderate	1.14*	(1.13–1.16)	1.14*	(1.11–1.16)	0.95*	(0.92–0.98)	0.95	(0.83–0.92)
Severe	1.33*	(1.31–1.35)	1.36*	(1.33–1.40)	0.85*	(0.82–0.88)	0.92*	(0.86–0.98)
Hypertension								
Yes	Reference							
No	1.08*	(1.06–1.09)	1.04*	(1.01–1.06)	0.84*	(0.81–0.86)	0.87*	(0.82–0.91)
Income level								
High	Reference							
Low	1.02	(1.00–1.05)	1.04	(1.00–1.09)	1.20*	(1.11–1.30)	1.56*	(1.33–1.82)
Middle	1.02	(1.00–1.05)	1.02	(0.98–1.07)	1.12*	(1.03–1.21)	1.36*	(1.17–1.59)

Abbreviations: CCI, Charlson Comorbidity Index; DPC, Diagnosis Procedure Combination. _e_β: exponentiated coefficients, percentage increase in mean cost per unit increase in the covariate. Significant values are shown with * *P* <.01.

 The DPC beds model shows that total hospitalization cost was not significantly associated with income levels; however, it was associated with the severity of CCI scores (moderate: _e_β = 1.14, 95% CI 1.13–1.16; severe: _e_β = 1.33, 95% CI 1.31–1.35), and the chronic diseases of hypertension (_e_β = 1.08, 95% CI 1.06–1.09). Significant decreases in total hospitalization cost were observed among patients aged 85 years and older. Total hospitalization cost was significantly increased with the presence of hypertension.

 In the results for the general beds model, the total hospitalization cost of different income levels was not significant. In addition, the total hospitalization cost increased with the presence of hypertension (_e_β = 1.04, 95% CI 1.01–1.06) and the increased severity of CCI scores (moderate: _e_β = 1.14, 95% CI 1.11–1.16; severe: _e_β = 1.36, 95% CI 1.33–1.40). Women were significantly associated with an increase in total hospitalization cost.

 In the medical chronic care beds model, a statistically significant increase in total hospitalization cost was associated with an increase in income levels (low-income: _e_β = 1.20, 95% CI 1.11–1.30; middle-income: _e_β = 1.12, 95% CI 1.03–1.21). Age category of 80–84 years old, and age category of 85 years and older demonstrated a statistically significant decrease in total hospitalization cost. However, total hospitalization cost was significantly decreased with increased severity of comorbidity conditions (moderate: _e_β = 0.95, 95% CI 0.92–0.98; severe: _e_β = 0.85, 95% CI 0.82–0.88). Similarly, total hospitalization cost was significantly decreased with the presence of hypertension (_e_β = 0.84, 95% CI 0.81–0.86).

 Lastly, in the psychiatric beds model, total hospitalization cost was significantly increased in the patients with low- and middle-income levels (low-income: _e_β = 1.56, 95% CI 1.33–1.82; middle-income: _e_β = 1.36, 95% CI 1.17–1.59) compared with high-income level. In addition, total hospitalization cost was significantly decreased with the appearance of hypertension (_e_β = 0.87, 95% CI 0.82–0.91). As observed, total hospitalization cost of psychiatric beds was significantly associated with severe comorbidity conditions (severe CCI: _e_β = 0.92, 95% CI 0.86–0.98).

###  Marginal Mean Effects of Income on Total Hospitalization Cost

 To evaluate the influence of income level on total hospitalization cost, the marginal means were estimated after generalized linear models (Table 5). In the utilization for 4 kinds of bed categories, total hospitalization cost decreased with the increase of income. With the increase of co-payment, total hospitalization cost decreased tremendously for medical chronical beds (low-income: 20 298, 95% CI 19 966–20 630; middle-income: 18 893, 95% CI 18 530–19 256; high-income: 16 926, 95% CI 15 646–18 207), and psychiatric beds (low-income: 21 624, 95% CI 20 963–22 285; middle-income: 18 947, 95% CI 18 221–19 674; high-income: 13 894, 95% CI 11 791–15 997).

**Table 5:** Marginal Effects of Income on Total Hospitalization Cost of Each Bed Categories (US$)

**Income Level**	**DPC Beds**		**General Beds**		**Medical Chronical Beds**		**Psychiatric Beds**	
	M*	**(95% CI)**	***M***	**(95% CI)**	***M***	**(95% CI)**	***M***	**(95% CI)**
Low	9121	(9038–9203)	8745	(8631–8860)	20 298	(19 966–20 630)	21 624	(20 963–22 285)
Middle	9102	(9025–9179)	8560	(8446–8673)	18 893	(18 530–19 256)	18 947	(18 221–19 674)
High	8914	(8680–9147)	8381	(8031–8730)	16 926	(15 646–18 207)	13 894	(11 791–15 997)

*M, Marginal mean—calculated based on the assumption that the value of each covariate is the mean.

## Discussion

###  Primary Findings

 This study revealed that hospitalization utilization was influenced by income levels, and varied greatly among each bed type. For DPC and general beds, hospitalization rates were comparatively higher than that for medical chronic care and psychiatric beds, despite their less total hospitalization cost and shorter LOS. The fact of this phenomenon might be that DPC and general beds undertake the function of acute term treatment, while medical chronic care and psychiatric beds mainly play a role in long-term care recovery, which tends to have longer LOS.^22-24^ Especially, when examining hospitalization status in DPC and general beds, total hospitalization costs were positively associated with hypertension and higher CCI score rather than income levels, indicating that patients’ health status greatly influence the use of acute treatment services. DPC beds and general beds are allocated mainly for patients with acute diseases, whereby patients would receive treatment according to the specified rules with limited treatment options, thus health status predicts healthcare utilization better than co-payment rate for acute treatment services.^25,26^ The marginal mean of total hospitalization cost for DPC and general beds only decreased slightly with the increase in income, which could further suggest that co-payment rate might have little impact on the use of acute treatment services.

 In contrast, when medical chronic care and psychiatric beds were examined, healthcare utilization for patients with low-income level was significantly higher than that for middle-, and high-income levels. Patients with low-income level could receive more benefits for hospitalization service with long-term care, such as medical chronic care and psychiatric beds care service, therefore, patients have greater intention to use hospitalization service.^27-29^ It is worth noting that total hospitalization cost is negatively associated with the CCI score. Consistent with the current findings, some studies suggested that older peoples’ healthcare utilization behaviour for long-term care facilities might be affected by some potential factors other than the severity of diseases.^30-32^ The marginal mean of total hospitalization cost for both medical chronic care and psychiatric beds were significantly decreased with the increase of income. A number of studies demonstrated that a higher co-payment would contribute to the decrease of long-term care medical costs, which could explain this result.^33-35^ To be specific, for psychiatric beds, the LOS is the longest among four bed types, and the total hospitalization cost decreased significantly with the increase of income level. The reason might be the policy inclination for lower income level, since the lower cap for out-of-pocket cost for lower income patients might encourage them to use hospitalization service once they have reached a certain amount of money,^27,28^ especially for those diseases related with long-term care service such as psychiatric conditions.^36,37^

###  Policy Implications

 A reasonable cost-sharing system must not only alleviate patients’ financial burden, but also control healthcare expenditure. Despite of the existence of co-payment rate policy and the cap for out-of-pocket cost, the overuse of healthcare services, especially among older adults, was prevalent, and was reflected in the phenomenon of “*social hospitalization*.”^10^ The insurance system for older adults was established in 1973 making healthcare services free of cost. A small co-payment was required in 1983 to address the increasing healthcare spending and the “*social hospitalization*” issues. After several changes, the current income-based co-payment rates for the late elderly people (high-income level: 30%; low- and middle-income level: 10%) were finalized in 2008.^38^ In addition, the policy of cap for out-of-pocket represents a subsidy for patients with high medical costs. This policy is commonly applied when an old patient, for example, suffers from severe diseases or requires expensive medical and long-term care.

 Although the Japanese government has attempted to address this so-called *“social hospitalization*” issue by increasing co-payment rate for the insured people in the last few decades, the positive effect of this co-payment on healthcare utilization was minimal. In fact, studies experimenting the effect of co-payment change in developed countries reported mixed findings.^39-42^ A study in Canada showed the result that the introduction of co-payment for the prescription drugs had resulted in more hospitalization events.^40^ Another research in the United States demonstrated that raising co-payment for elderly patients might unexpectedly increase total spending on healthcare and extend the hospital stays.^41^ However, the study in Germany reported that the elimination of the co-payment did not change the hospitalization frequency.^39^ A recent study in Japan found that the increase of co-payment rate for insured individuals could decrease insurer’s payment, albeit it would increase the total spending on healthcare.^42^

 Our findings showed that there were a substantial number of cases of elderly patients stayed in hospital for an extremely long period. This long hospital stays could be attributed to actions by healthcare providers as well as patients. Healthcare providers would welcome the use of long-term care services, because the longer a patient stays in the hospital, the more profits are generated. On the other hand, patients also prefer to choose nursing care in hospitals as a substitute for long-term care because of its perceived quality and a relatively low out-of-pocket cost for the low-income patients. Thus, the current income-based cap policy might induce excessive healthcare use, especially among economically disadvantaged patients. A few studies reported that a low cap could substantially increase the use of hospital admission and the LOS.^43-45^ The issues related to the “*social hospitalization,*” to a limited extent, could be addressed through the revision of cap. Increasing the current co-payment cap to a relative higher amount, while maintaining the current co-payment rate, might offer a promising solution to discourage patients from using hospital admission service as a substitute for long-term elderly care. Therefore, relevant stakeholders including representatives from insurance organizations, healthcare providers, health economists, and relevant academics are encouraged to work together to determine the feasibility of this insurance design and its mechanisms, together with our proposed revision of co-payment cap or possible method to control healthcare spending.

 A number of studies demonstrated that the value-based insurance programs adopted by other countries could successfully reduce the length of hospital stay and lower healthcare spending.^46-48^ However, given the inherent uniqueness and the complexities of the Japanese healthcare system, decision to adopt such a program would require an extensive study. On a related note, Japan is experiencing a transition from hospital-based healthcare services to community-based healthcare services;^49^ however, a smooth transition could only be achieved if such a plan sufficiently gains public support. Patients might perceive the quality of care received in a hospital is far more superior than similar services received from community-based healthcare facilities or long-term nursing facilities. Therefore, public campaigns regarding the need for sustainable community-based healthcare services could provide a good opportunity to increase people’s awareness, and consequently support the utilization of community-based healthcare services in Japan.^50^ At the government level, an appropriate hospital admission guideline must be communicated and issued to all hospitals. This guideline must consist of strict hospital admission criteria that must be fulfilled before a specific patient admission is permitted. To ensure hospital adherence to this guideline, the audit process must be regularly done, and perhaps financial incentives could be introduced to reward the performing hospitals. Nonetheless, an enhanced collaboration between hospitals, long-term care facilities and other entities providing community-based healthcare services is also needed enabling medical resources and expertise to be shared. With this inter-collaborative effort, improvement of public perception regarding the quality of the long-term care facility is anticipated.

###  Limitations

 This study is subject to some methodological limitations. One limitation is that we were unable to ascertain which bed category was utilized if a patient had exceeded the co-payment cap. Therefore, we calculated the actual average cost-sharing percentage for each income level, and the analysis was based on the assumption that patients consider the actual average cost-sharing percentage as their co-payment rate. For this reason, the results might provide only a rough estimation of marginal effect of co-payment when the co-payment cap was exceeded, especially for patients with long-term care services. We, nevertheless, analyzed total hospitalization cost based on hospitalization bed categories to describe the current utilization of hospital beds, at the same time provide an avenue for discussion of cost-sharing policy improvement in Japan.

 In addition, issues related to the confounding in comorbidity status (CCI scores) and social status (income) data—when the data were regressed together—might result in confounding errors. This issue is unavoidable due to the nature of the data we have. Despite our best intention to address the issues using sophisticated methods such as using discontinuity regression and difference-in-difference analyses, we could not perform such analyses due to data limitation. However, examinations of correlation between variables revealed the effect size of correlation was rather small despite being significant. Further examination showed the variance inflation factor values were less than 5.0, indicating no significant issues with multicollinearity. Thus, we have reasons to believe the confounding effects—if present—would be minimal.

 Despite all these limitations, the claims data from the healthcare insurance database covered more than 600 000 insured people, which may ensure a large-enough sample to obtain robust findings. However, to better analyze such data, we recommend researchers to explore more sophisticated design methods, such as discontinuity regression and difference-in-difference analysis, to overcome the limitations faced by the current study.

## Conclusion

 The current cost-sharing policies have created potential incentives for hospitals and patients to occupy beds as long as possible to receive more benefits. A lower cap for out-of-pocket could provide a promising solution to suppress the use of long-term hospital service. Public campaigns could be regularly organized to increase people’s awareness of need for sustainable community-based care services. The collaboration between hospital and community-based care facilities should be enhance to develop the quality of long-term care services.

## Acknowledgements

 The authors thank the Wide-Area Association of Latter-Stage Elderly Healthcare of Fukuoka Prefecture for their provision of a healthcare claims database. We thank Peter Fogarty, MA English 1st Class, from Edanz Group (www.edanzediting.com/ac), for editing a draft of this manuscript.

## Ethical issues

 The Institutional Review Board of Kyushu University (Clinical Bioethics Committee of the Graduate School of Medical Sciences, Kyushu University) approved this study. We used anonymized claims insurance data, therefore, the requirements for obtaining inform consent was not required.

## Competing interests

 Authors declare that they have no competing interests.

## Authors’ contributions

 YL: conception and design, acquisition of data, analysis and interpretation of data, drafting the manuscript and statistical analysis. AB: conception and design, critical revision of the manuscript for important content, supervision, and administrative and material support. AJ: conception and design, analysis and interpretation of data, and critical revision of the manuscript for important content. PJ: conception and design, and acquisition of data. TF: revision of manuscript for important content.

## Authors’ affiliations

^1^Department of Health Care Administration & Management, Graduate School of Medical Sciences, Kyushu University, Fukuoka, Japan. ^2^Health Administration Program, Faculty of Business & Management, Universiti Teknologi MARA, Selangor, Malaysia. ^3^Department of Health Sciences, Faculty of Medical Sciences, Kyushu University, Fukuoka, Japan.
